# Physiological Imaging Methods for Evaluating Response to Immunotherapies in Glioblastomas

**DOI:** 10.3390/ijms22083867

**Published:** 2021-04-08

**Authors:** Sanjeev Chawla, Vanessa Shehu, Pradeep K. Gupta, Kavindra Nath, Harish Poptani

**Affiliations:** 1 Department of Radiology, Perelman School of Medicine at the University of Pennsylvania, Philadelphia, PA 19104, USA; Vanessa.Shehu12@gmail.com (V.S.); Pradeep.Gupta@pennmedicine.upenn.edu (P.K.G.); Kavindra.Nath@pennmedicine.upenn.edu (K.N.); 2 Department of Molecular and Clinical Cancer Medicine, University of Liverpool, Liverpool L69 3BX, UK

**Keywords:** glioblastoma, immunotherapy, treatment response, diffusion MR imaging, perfusion MR imaging, positron emission tomography

## Abstract

Glioblastoma (GBM) is the most malignant brain tumor in adults, with a dismal prognosis despite aggressive multi-modal therapy. Immunotherapy is currently being evaluated as an alternate treatment modality for recurrent GBMs in clinical trials. These immunotherapeutic approaches harness the patient’s immune response to fight and eliminate tumor cells. Standard MR imaging is not adequate for response assessment to immunotherapy in GBM patients even after using refined response assessment criteria secondary to amplified immune response. Thus, there is an urgent need for the development of effective and alternative neuroimaging techniques for accurate response assessment. To this end, some groups have reported the potential of diffusion and perfusion MR imaging and amino acid-based positron emission tomography techniques in evaluating treatment response to different immunotherapeutic regimens in GBMs. The main goal of these techniques is to provide definitive metrics of treatment response at earlier time points for making informed decisions on future therapeutic interventions. This review provides an overview of available immunotherapeutic approaches used to treat GBMs. It discusses the limitations of conventional imaging and potential utilities of physiologic imaging techniques in the response assessment to immunotherapies. It also describes challenges associated with these imaging methods and potential solutions to avoid them.

## 1. Introduction

Glioblastoma (GBM) is a devastating and universally fatal brain cancer [1]. The current standard of care for GBM comprises maximal safe surgical resection followed by concurrent chemoradiation therapy (CCRT) and maintenance chemotherapy with temozolomide (TMZ). Despite multimodal treatment, prognosis remains dismal with a median overall survival (OS) of 14–16 months from initial diagnosis [1]. Because of the aggressive and infiltrative nature of GBMs, tumor recurrence is inevitable after initial therapy [2]. At recurrence, treatment options are limited with no standard approach being established, and patients may be treated with repeat surgery, reirradiation, chemotherapy, tumor treating fields or antiangiogenic therapy [3,4]. However, these interventions largely remain palliative and are associated only with partial response and variable survival benefits [3]. There is hence a pressing need for the development of novel and more effective therapeutic strategies for GBMs.

In the quest for an effective treatment, several immunotherapeutic approaches have been introduced in recent years that have been designed to harness patient’s immune response to fight and eliminate tumor cells. Broadly, these novel strategies can be divided into four major classes: immunomodulators, active immunotherapy, adoptive immunotherapy, and oncolytic viral therapy [5,6,7,8,9,10,11]. Although immunotherapy has yet to be established for providing consistent clinical benefits in GBM, several immunotherapy trials have reported acceptable safety profiles and survival benefits in small cohorts of patients [12,13,14,15].

It has been reported that patients treated with immunotherapy demonstrate profound inflammation at the tumor sites, often referred to as treatment-induced pseudoprogression (PsP), which can suggest a favorable treatment outcome [16]. Unfortunately, standard clinical MR imaging is unable to distinguish true progression (TP) from PsP [7,17,18]. It is thus imperative to develop robust, reliable and reproducible imaging methods that can provide accurate assessment of treatment response. Since immunotherapies can result in delayed responses, imaging methods can prevent responsive patients from discontinuing a possibly beneficial treatment and similarly can aid non-responsive patients from continuing a potentially harmful and ineffective treatment. Physiologic imaging methods such as diffusion and perfusion imaging as well as amino acid and reporter gene-based positron emission tomography (PET) provide valuable information about tumor biology and microenvironment [19,20,21]. Several studies [22,23,24,25,26,27,28,29,30,31] have reported the potential of these imaging techniques in the evaluation of treatment response to CCRT and antiangiogenic therapies in GBM patients, suggesting that these techniques can also aid in assessing treatment response to immunotherapies.

This review is structured into three sections to cover the role of imaging in immunotherapy of GBMs. The first section covers commonly used immunotherapeutic approaches used to treat GBM patients. The second section discusses the limitations of conventional imaging methods to emphasize the need for alternative imaging techniques in the evaluation of treatment response to immunotherapies. Finally, in the third section, potential applications of physiologic MR and PET imaging methods are described for the assessment of immunotherapies in GBMs. The challenges associated with these imaging methods and possible solutions to avoid those pitfalls have also been described.

## 2. Immunotherapeutic Approaches for Glioblastomas

For several years, it was assumed that the brain lacks a lymphatic system since it was considered an immuno-privileged organ (devoid of any immune cells). However, a strong body of evidence [32,33,34] has demonstrated the presence of a lymphatic/glymphatic system inside the brain through which the brain interacts with the peripheral immune system. This finding overturned the prevailing dogma such that the brain is now considered as an immunocompetent organ, and it also prompted renewed enthusiasm for immunotherapies in the treatment of brain tumors. Nevertheless, the development of immunotherapeutic approaches against GBM faces several challenges. Firstly, GBMs are considered to be immunosuppressive tumors due to several factors including lymphopenia driven by bone marrow suppression, low tumor mutational burden, overexpression of transforming growth factor (TGF)-β and upregulated cell populations of protumor and anti-inflammatory tumor-associated macrophages (M2), as well as regulatory T cells (Tregs), which collectively facilitate tumor cells to escape immune surveillance [35]. Secondly, the use of radiation therapy and TMZ (an alkylating agent) further enhances this immunosuppressive mechanism. Furthermore, steroids that are typically administered to GBM patients for management of peritumoral edema are known to decrease the efficacy of immunotherapies [36]. To overcome the highly immunosuppressive tumor microenvironment in GBMs, inhibiting the activity of histone deacetylases has been proposed [37]. Despite these challenges, immunotherapeutic strategies that were initially considered irrelevant are now being actively pursued to determine their potential in improving the clinical outcomes of GBM patients. 

**Figure 1:**
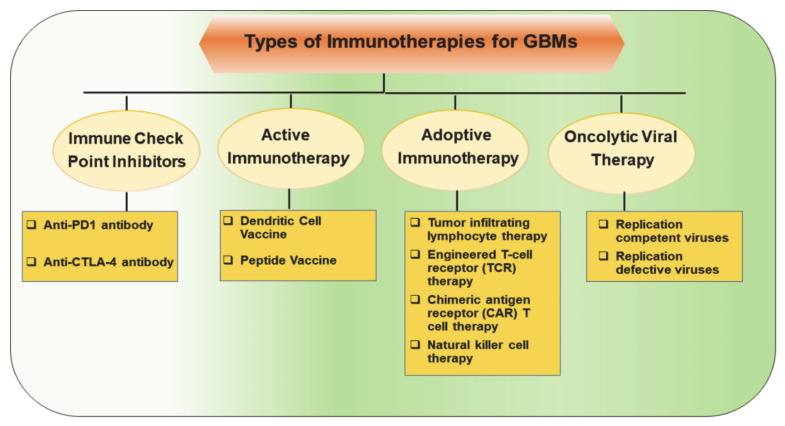
Major classes of immunotherapeutic approaches to harness patient’s immune response against tumor cells in glioblastomas (GBMs).

Several promising novel immunotherapeutic approaches are being actively investigated in clinical trials for GBM treatment (Figure 1) [7,8,9,10,11]. These include inhibitors of immune checkpoint regulators, antitumor vaccinations, adoptive transfer of genetically modified and cytotoxic T-lymphocytes, and generation of genetically engineered oncolytic viruses.

Essentially, these studies have been conducted to test the feasibility, to establish safety profile and to evaluate the therapeutic efficacy of novel immunotherapies in a diverse population of GBM patients. However, the food and drug administration (FDA) has not yet approved any immunotherapeutic approach for the treatment of GBM despite the completion of several clinical trials, probably due to the variability in the success rate achieved with these therapies.

In the following sections, a brief overview of commonly used immunotherapeutic approaches currently used in treating GBM patients is described. In particular, the action mechanism, safety profile and therapeutic efficacies of major classes of immunotherapies are discussed. Readers are referred to excellent articles available for a detailed overview on immunotherapies in GBMs [11,38,39].

### 2.1. Immune Checkpoint Inhibitors (Immunomodulators)

Immune checkpoint inhibitor (ICI)-based immunotherapy has revolutionized the treatment of several cancers, including metastatic melanoma [40], non-small cell lung cancer [41] and renal cell carcinoma [42]. Using ICIs, improved survival outcomes were demonstrated in a murine model of gliomas, suggesting that monoclonal antibodies can also be used for the treatment of GBM [43]. As such, ICIs (immunomodulators) are currently at the forefront of immunotherapeutic strategies under investigation for the treatment of GBMs.

#### 2.1.1. Mechanism of Action

**Figure 2:**
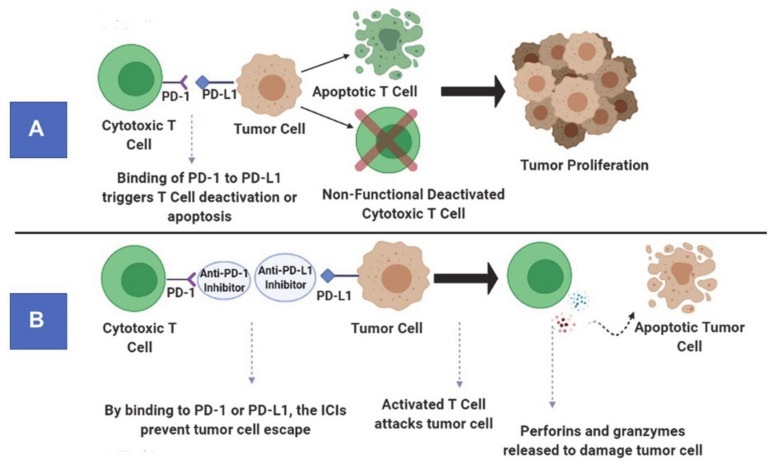
Principle of immune checkpoint pathways under physiological conditions (**A**). Mechanism of action of anti-programmed cell death protein-1 (PD1) antibody (an immune checkpoint inhibitor) (**B**).

Under physiological conditions, checkpoint pathways play a critical role in maintaining immune homeostasis by inhibiting the proliferation, activity and responsiveness of cytotoxic T-lymphocytes (Figure 2A). While this is important for attenuating autoimmunity, it helps tumor cells in evading the immune system. As the ICIs are monoclonal antibodies, they prevent tumor cells from suppressing the activity of cytotoxic T cells. These monoclonal antibodies block immune checkpoint proteins present on T cells from binding to inhibitory ligands present on tumor cell surfaces (Figure 2B). The immunoreceptor proteins are present on the surfaces of T cells and bind to their respective ligands on antigen-presenting cells (APCs) to downregulate immune system activity [44]. In GBMs, inhibitory checkpoint proteins are frequently upregulated, whereas stimulatory checkpoint proteins are downregulated [45]. The key players in the inhibitory checkpoint signaling pathways include programmed cell death protein-1 (PD1) and cytotoxic T-lymphocyte-associated protein-4 (CTLA-4). While PD1 is expressed on activated B cells, T cells, natural killer cells, and myeloid cells, its ligands (PD-L1 and PD-L2) are upregulated in activated leukocytes, myeloid cells and tumor cells. On the other hand, CTLA-4 is expressed on T cells, and its ligands (CD80 and CD86) are expressed on the surface of APCs. By disrupting the checkpoint regulatory pathways, ICIs stimulate cytotoxic T-lymphocyte mediated tumor cell killing, reduce the population of Tregs and cause the production of cytokines resulting in a profound inflammatory response within the tumor bed [46]. 

#### 2.1.2. Safety Profile and Therapeutic Efficacy

**Figure 3:**
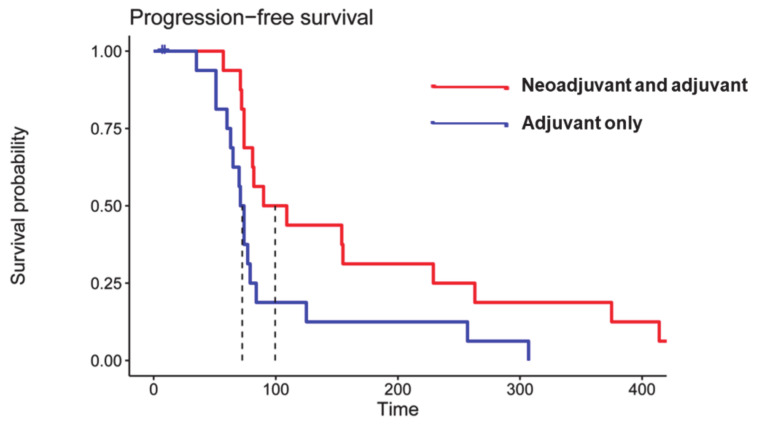
Kaplan–Meier curves showing significantly prolonged progression-free survival (PFS) in patients who received neoadjuvant and adjuvant pembrolizumab (median PFS = 72.5 days, red curve) compared to patients who received pembrolizumab only in the adjuvant setting (blue curve) (median PFS= 99.5 days, two-sided p = 0.03 by log-rank test). Reprinted with permission from ref. [49]. Copyright 2019 Springer Nature America, Inc.

Some ICIs such as pembrolizumab (anti-PD1 antibody), nivolumab (anti-PD1 antibody), and ipilimumab (anti-CTLA-4 antibody) have been used for the treatment of GBMs in several large multicentric early- and late-phase clinical trials [11,34,47,48,49]. While some phase I/II trials have established the tolerability and safety profile of multiple ICIs in GBM patients, other studies [50] have revealed that anti-PD1 immunotherapy is associated with less toxicity and side effects than anti-CTLA-4 antibodies. While exploring the therapeutic efficacy of anti-PD1 immunotherapy, the results from a phase III trial have revealed that nivolumab does not improve the OS or progression-free survival (PFS) compared to bevacizumab in recurrent GBM patients [51]. However, a recent randomized, multi-institutional clinical trial reported that patients treated with pembrolizumab had significantly prolonged OS and PFS (Figure 3) [49]. Moreover, considerable upregulation of T cell and interferon-γ-related gene expression was observed within tumor specimens following pembrolizumab treatment. Currently, there are several ongoing clinical trials whose results are yet to be published.

Some investigators have also suggested the use of bevacizumab (an antivascular agent) as a steroid substitute to control peritumoral edema and to avoid immunosuppressive actions of steroids. While preliminary results from these studies support the safety of adding bevacizumab to immunotherapies, clinical benefits have not yet been established [11,52].

### 2.2. Active Immunotherapy 

Active immunotherapy exploits the use of neoantigens that are overexpressed (tumor-associated antigens) or exclusively expressed (tumor-specific antigens) by tumor cells to train the host’s immune system to target tumor cells and in turn inhibit tumor growth. This type of immunotherapy elicits an immune response by presenting tumor antigens towards major histocompatibility complexes (MHCs), which allows T cells to bind, multiply and target tumor cells to be destroyed [53]. Various types of vaccine immunotherapies such as tumor cell-based, dendritic cell-based, peptide-based and genetic-based vaccines are being tested in various neuro-oncology clinical trials.

#### 2.2.1. Mechanism of Action

Dendritic cells (DCs) are potent immune stimulators that can prime and activate T cells in several organs. These primed T cells have additional potential to mount secondary memory responses. Autologous DCs are differentiated from harvested monocytes, cultured and loaded with antigens (whole tumor cell lysate) from the patient’s tumor specimens obtained at surgical resection. These modified DCs are then treated with a differentiation factor such as granulocyte-macrophage colony-stimulating factor and finally administered back to the patients intradermally. Rindopepimut is a vaccine that has been widely used in the treatment of GBMs [38,39]. This is a peptide vaccine that targets a mutant protein known as epidermal growth factor receptor deletion mutation (EGFRvIII), present in about 25–30% of GBMs. Structurally, rindopepimut consists of a 13-amino acid peptide conjugated to a non-specific immunomodulator keyhole limpet hemocyanin segment [54].

#### 2.2.2. Safety Profile and Therapeutic Efficacy

A systematic review by Liau et al. [55] revealed that DC vaccines are associated with an acceptable tolerability profile, limited capacity for delaying tumor recurrence and modest survival benefits in GBM patients. In a randomized, international phase III trial, rindopepimut, a vaccine targeting the EGFRvIII, was evaluated in newly diagnosed EGFRvIII^+^ GBM patients [56]. Despite the presence of strong humoral responses to the vaccine, rindopepimut did not increase survival outcomes in patients compared to controls. Interestingly, a number of patients who did not receive the active vaccine and underwent repeat surgery during the trial had lost EGFRvIII expression. This finding underscores the fact that heterogeneity of antigen expression in GBM is a major obstacle to the success of monovalent vaccines. 

### 2.3. Adoptive Immunotherapy

**Figure 4:**
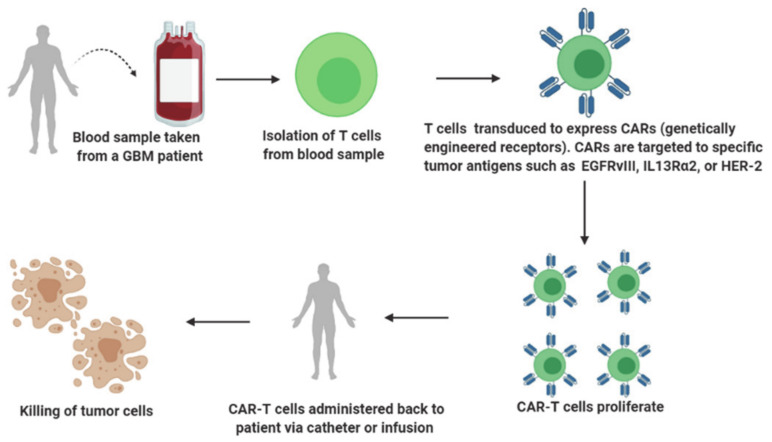
A brief overview of adoptive immunotherapy.

Adoptive immunotherapy, also known as cell-based immunotherapy, is a form of treatment that activates, enhances and expands the population of tumor-specific T cells prior to reinfusing them back into the patient’s body (Figure 4). Adoptive immunotherapy can be classified into (a) tumor-infiltrating lymphocyte therapy, (b) engineered T-cell receptor (TCR) therapy, (c) chimeric antigen receptor (CAR) T cell therapy and (d) natural killer cell therapy. Adoptive immunotherapies that selectively target tumor cells while leaving normal cells unharmed have evolved considerably over the years and are in different stages of their advancement for GBM treatment. However, the most promising adoptive immunotherapy involves T cells redirected with CARs targeting the B cell marker CD19 that has revolutionized the treatment of hematological malignancies such as acute lymphoblastic leukemia, chronic lymphocytic leukemia and large diffuse B cell lymphomas [57,58,59,60]. 

#### 2.3.1. Mechanism of Action

In order to elicit an immune response, the T cell requires (a) T cell receptors (TCRs) that recognize and bind to the antigen peptides present on MHCs exposed on the surface of APCs or tumor cells and (b) co-stimulatory signaling molecules such as CD28, 4-1BB and OX40. These molecules bind to the ligands expressed on APCs or tumor cells. Compared to conventional TCRs, CARs are synthetic, genetically engineered receptors that can target surface molecules in their native conformation. Structurally, a CAR molecule consists of an extracellular, antigen-recognizing and binding domain, which is usually a single-chain variable fragment (scFv) [61]. This design not only enables the recognition of a broad array of antigens but also obviates the need for presentation of antigens by MHCs. The loss of MHC class-I expression in tumor cells is a common mechanism leading to tumor escape and resistance to T cell immunity [62]. The fact that CAR T cells can recognize and bind to tumor antigens unrestricted to MHC class I expression makes CAR T cell therapy an attractive approach for the development of anti-tumor therapies.

The extracellular domain of CAR is linked through a hinge and spacer present on the transmembrane domain and an intracellular signaling domain. While first-generation CAR construct contains CD3ζ in isolation in the intracellular domain, second- and third-generation constructs include CD3ζ as well as one or two co-stimulatory domains such as CD28, OX40 and 4-1BB respectively [63]. Compared to the first generation, the second- and third-generation CAR T cells have shown improved proliferation and effector functions [64]. The fourth generation of CAR T cells is composed of additional genetic modifications that allow the release of transgenic proteins such as cytokines to enhance the immunogenicity of CAR T cells [65]. 

#### 2.3.2. Safety Profile and Therapeutic Efficacy

While CAR T cell therapy has shown marked and durable efficacy in hematologic tumors, it has not demonstrated desirable efficacy in solid tumors, including GBMs. Lately, Brown et al. [15] reported complete regression of all intracranial and spinal tumors in a patient with recurrent multifocal GBM who received multiple infusions of CAR-engineered T cells targeting the tumor-associated antigen interleukin-13 receptor alpha-2 (IL13Rα2). The positive response continued for 7.5 months after the initiation of CAR T cell therapy, and this case report highlights the therapeutic potential of CAR T cells in GBM. Using a different immunogenic target, a recent study [66] demonstrated successful synthesis, delivery and acceptable safety profile of CAR T cell therapy targeting against EGFRvIII epitope in a cohort of 10 patients with recurrent GBM who received a single dose of therapy. All treated patients had detectable amounts of EGFRvIII CAR T cells in the peripheral blood. Additionally, available evidence suggested that CAR T cells had effectively trafficked within the active regions of tumors. Although the study was not designed to assess therapeutic efficacy, one patient had stable disease lasting >34 months. Collectively, these studies provide initial evidence of safety and antitumor activity of CAR T-cell immunotherapy in GBM patients. 

### 2.4. Oncolytic Viral Therapy

Oncolytic viral immunotherapy is based upon utilization of either live, immunogenic, or replication-competent viruses that selectively infect and replicate inside the tumor cells to stimulate immune response within the tumor microenvironment or utilization of replication-defective viruses that deliver anti-tumor genes to tumor cells but do not replicate [53,67]. Virotherapy is fast emerging as a special type of immunotherapy that is administered intravenously or peritumorally through the resection cavity or intratumorally through a stereotactic-guided catheter by means of convection-enhanced delivery.

#### 2.4.1. Mechanism of Action

Natural viruses are genetically engineered so that they can selectively replicate only inside the tumor cells without infecting the surrounding normal healthy cells. When these replication-competent viruses infect tumor cells and replicate, they lead to tumor cell cytotoxicity and spread the infection to nearby tumor cells. As a result, innate immune cells such as macrophages get activated and release cytokines, triggering intense immune response within the tumor microenvironment. Lysis of tumor cells enhances antigen presentation and thereby increases recruitment of activated effector T cells against invading tumor and viral antigens, which leads to durable antitumor response [68]. On the other hand, replication-defective viruses such as adenoviral vectors are modified to deliver the therapeutic gene, such as the HSV-tk gene. Once inside the tumor cell, the adenoviral DNA exists as an extrachromosomal element in the nucleus and transcribes/translates the thymidine kinase gene. Through a subsequent cascade of reactions, this mechanism leads to DNA replication arrest within the tumor cell, ultimately culminating in immunogenic tumor cell death [68].

#### 2.4.2. Safety Profile and Therapeutic Efficacy

Most early phase clinical trials using oncolytic viruses including adenovirus, HSV, poliovirus, measles virus, parvo virus and retrovirus have been tested in recurrent GBMs. In general, these trials have demonstrated feasibility and acceptable safety of oncolytic viral immunotherapy but have not reported substantial efficacy in terms of survival benefit [69,70]. However, preliminary results from other clinical trials have demonstrated remarkable efficacy and survival benefits only in subsets of patients [71]. Incredibly, a clinical trial employing a recombinant poliovirus for the treatment of recurrent GBM reported significant survival benefits with 3-year OS rate of 21%, whereas patients in the control group had a 3-year OS rate of only 4% [72]. Additionally, some promising strains of infectivity-enhanced, replication-competent and tumor-selective viruses such as DNX-2401, PVSRIPO and Toca 511 have shown durable responses in approximately 20% of GBM patients who received the viruses intratumorally [72,73,74]. 

Oncolytic viruses can also be used to transfer therapeutic payloads to tumors [75]. Viruses equipped with immunoregulatory inserts such as interleukin-12 and OX40 ligand have been tested in clinical trials. Other examples of clinically tested viruses with therapeutic payloads include gamma retrovirus (Toca 511) and vaccinia virus (TG 6002) carrying cytosine deaminase (CD) gene. When active in infected tumor cells, CD can convert the subsequently administered 5-fluorocytosine (5-FC) drug into chemotherapeutic fluorouracil [76]. However, results from a completed phase I/II clinical trial investigating the safety and efficacy of Toca 511 plus 5-FC as a treatment modality for recurrent GBMs are still awaited.

## 3. Standard Clinical Neuroimaging Methods for Response Assessment to Immunotherapy

Clinical MR imaging is the mainstay in assessing treatment response to therapy in neuro-oncology. Patterns of contrast enhancement on post-contrast T1-weighted images and/or extent of hyperintense signal-intensity on T2-FLAIR images are typical features that are used to evaluate treatment response in GBM patients. However, these morphological features reflect only impairment in the integrity of blood–brain barrier (BBB) or the extent of edema and are thus nonspecific, as can be observed both in TP and PsP, making their distinctions almost impossible in most situations. The criteria for the assessment of treatment response in high-grade gliomas have been proposed by the response assessment in the neuro-oncology (RANO) working group [77]. These include measurement of enhancing lesions via 2D-biperpendicular diameter of the enhancing region as the basis of response, but also incorporate qualitative evaluation of T2-FLAIR abnormality. Updated RANO criteria require a minimum period of 12 weeks after the completion of CCRT and a repeat scan after 4 weeks for confirmation of TP unless the site of progressive disease is distant from the radiation field or there is pathologic evidence of TP [77].

Since immunotherapy-specific response criteria should also account for delayed responses that are often observed with immunotherapies, the RANO committee further redefined the response assessment criteria for patients with GBM undergoing immunotherapy and coined the term iRANO, which was specifically designed for evaluating response to immunotherapy of GBMs [78]. According to iRANO criteria, patients harboring a lesion at the site of the original tumor and presenting with a possible diagnosis of TP or even presenting new lesions at distant sites within the first 6 months of the commencement of immunotherapy should be continued with treatment. Furthermore, confirmation of radiographic progression should be performed at 12 weeks instead of 4 weeks after the initial neuroimaging assessment of TP disease [78]. Given that GBM patients have a short life expectancy, and patients with suspected TP have to wait for 12 weeks while receiving potentially non-effective immunotherapy, the iRANO criteria may adversely impact the clinical management of a TP patient. Moreover, subjective interpretation of the iRANO criteria is somewhat controversial amongst neuroradiologists in assessing treatment response. Additionally, differences in head tilt and accurate identification of longest and perpendicular diameters, especially when the enhancing lesion has an irregular shape and/or ill-defined boundaries, further complicates the assessment of therapeutic response. 

To address these shortcomings and to find more reliable MR imaging features, a novel approach using high-resolution treatment response assessment maps (TRAMs) has been proposed to determine treatment outcomes in GBM patients treated with CCRT [79] and bevacizumab [80]. A similar approach was also used in a pilot study while evaluating treatment response to DC-based immunotherapy in GBM patients [7]. The premise of TRAM is based on the acquisition of two high-resolution 3D-T1-weighted images, one set of images at 3–5 min following the injection of gadolinium-based contrast agents and a second set acquiring with a delay of >1-h postcontrast. Subsequently, these images are subtracted and color-coded to represent the spatial distribution of contrast accumulation (red regions) and clearance (blue regions). Active tumor regions/TP with viable and undamaged blood vessels on TRAMs signify effective clearance of gadolinium-based contrast agent, whereas treatment effects/PsP with necrotic and occluded blood vessels represent contrast accumulation. TRAMs maps are relatively simple to acquire, readily interpretable and also not sensitive to susceptibility artifacts. Though promising, this method is associated with limitations including the requirement to wait >1-h after contrast injection, which makes it challenging from a workflow standpoint in busy academic medical centers. Moreover, the timings of post-contrast acquisitions are particularly important for correct interpretation. 

Currently, both iRANO criteria and the TRAM approach have not been prospectively validated for use in GBM immunotherapy trials and remain under research investigation. As immune-related response criteria continue to evolve, additional research is focusing on the development of physiologic imaging markers to evaluate treatment-related changes in the early window of post immunotherapy.

## 4. Role of Physiologic MR and PET Imaging in the Assessment of Treatment Response to Immunotherapies

Contrary to conventional anatomic MR imaging methods, physiologic imaging techniques, such as diffusion and perfusion MR imaging is more sensitive to biophysical processes within the tissues and hence provide more comprehensive information about the tumor microenvironment, including tumor cellular proliferation, organization of tumor cells, tumor hemodynamics and vascular permeability [81]. These techniques have been successfully integrated with standard MR imaging acquisition protocols at many institutions around the world. 

Readers are referred to excellent review articles about these methods with specific applications to neuro-oncology [21,82,83]. In brief, analysis of diffusion MR imaging data provides several parameters such as apparent diffusion coefficient (ADC), mean diffusivity (MD), fractional anisotropy (FA), coefficient of linear (CL), planar (CP) and spherical (CS) anisotropies. However, these parameters are influenced by several factors such as temperature, viscosity, cellular density, cell membranes and the presence of intracellular organelles along with macromolecules within the biological tissues [84]. Both MD and ADC are comparable and provide similar information about the degree of water diffusivity. The FA signifies the degree of directionality of water movement within a voxel, and its value ranges from 0 (isotropic) to 1 (maximally anisotropic). Other parameters (CL, CP and CS) describe the shape of a diffusion tensor that is related to macroscopic organization of tumor cells [85]. 

Perfusion MR imaging-data-derived parameters include cerebral blood volume (CBV), an index of capillary bed density and volume transfer constant (K^trans^), which is a measure of vascular permeability and blood flow. Both parameters are also putative markers of angiogenesis. Other clinically important parameters derived from perfusion MR imaging methods include volume fraction of extravascular-extracellular space in tissue (v_e_) which is inversely correlated to cellularity and mitotic activity and volume fraction of plasma space in tissue (v_p_), reflecting angiogenic activity in tumors [86].

Over the last several years, [^18^F]-2-Fluoro-2-deoxy-D-glucose ([^18^F]-FDG) has been the most commonly used PET tracer to study brain tumors [87]. Elevated [^18^F]-FDG uptake in neoplastic cells reflects increased expression of glucose transporters and/or enzymatic activity of hexokinase [88]. Numerous studies [89,90] have shown the potential of [^18^F]-FDG in studying metabolic activity in different types of cancers. However, normal brain parenchyma has naturally high uptake of [^18^F]-FDG, which undermines the diagnostic accuracy of FDG tracer for accurate delineation of brain tumor margins, especially from adjacent gray-matter regions. On the other hand, several amino acid-based PET imaging tracers have emerged as alternative candidates for metabolic imaging of brain tumors [87,91]. These tracers are characterized by high tumor-to-brain contrast based on their relatively high specificity for neoplastic cells and low accumulation in normal brain tissues. Frequently used amino acid tracers include O-(2-[^18^F]fluoroethyl)-L-tyrosine ([^18^F]-FET), [^11^C]methyl-L-methionine ([^11^C]-MET), and 3,4-dihydroxy-6-[^18^F]fluoro-L-phenylalanine ([^18^F]-FDOPA) targeting energy independent amino acid transporters of L-type (LAT) that are known to be upregulated in brain tumors [92,93]. In addition to amino acid-based tracers, ^18^F-fluorothymidine ([^18^F]-FLT), a pyrimidine analog, has been studied as a surrogate marker for cellular proliferation in neuro-oncology because of its preferential uptake by rapidly dividing neoplastic cells where it indicates the activity of thymidine kinase-1 (a key enzyme involved in DNA synthesis) [94].

**Figure 5:**
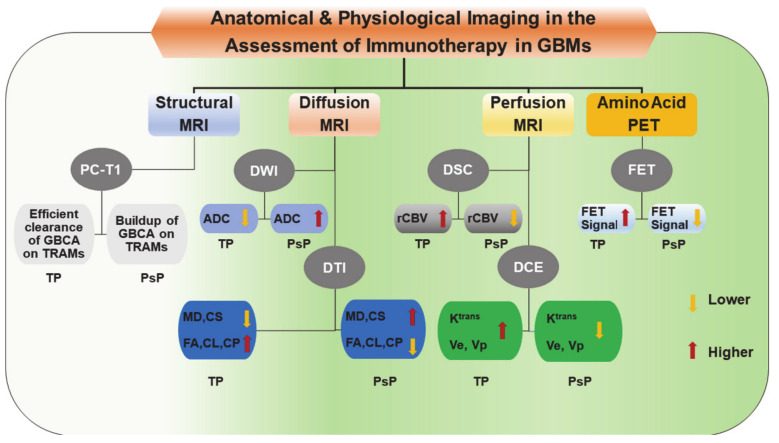
A block diagram is showing trends in structural, and physiologic imaging parameters that are usually observed in distinguishing true progression (TP) from pseudoprogression (PsP) in GBMs following treatment with immunotherapy. ADC, Apparent diffusion coefficient; CL, Coefficient of linear anisotropy; CP, Coefficient of planar anisotropy; CS, Coefficient of spherical anisotropy; DCE, Dynamic contrast-enhanced; DSC, Dynamic susceptibility contrast; DTI, Diffusion tensor imaging; DWI, Diffusion weighted imaging; FA, Fractional anisotropy; FET, O-(2-[^18^F] fluoroethyl)-L-tyrosine; GBCA, gadolinium-based contrast agent; GBM, Glioblastoma; K^trans^, Volume transfer constant; MD, Mean diffusivity; MRI, Magnetic resonance imaging; PC-T1; Post-contrast T1 weighted images; PET, Positron emission tomography; PsP, Pseudo-progression; rCBV, relative cerebral blood volume; TP, True Progression; TRAM, Treatment response assessment map; Ve, Fraction of extracellular-extravascular space; Vp, Fraction of plasma volume.

In the subsequent sections, an overview of the potential utility of these physiologic MR imaging as well as PET imaging techniques in the evaluation of treatment response to immunotherapies in GBM is provided. The general trends in structural and physiologic imaging-derived parameters in distinguishing TP from PsP in GBMs treated with immunotherapies are shown in Figure 5.

### 4.1. Checkpoint Inhibitors

Using physiologic MR imaging metrics, some studies [95,96] evaluated the response to anti-PD1-L1 therapy in recurrent GBMs. In one retrospective study [96], temporal changes in diffusion and perfusion MR imaging-derived parameters were investigated to evaluate treatment response. Using the last MR imaging at 6 months or beyond (mean duration of 7.8 ± 1.4 months from baseline) from the start of immunotherapy, and using the modified RANO criteria [97], the investigators observed that the interval change in relative ADC (rADC) values before and after treatment (mean interval time = 2.7 ± 1.0 months) was indicative of treatment response. A majority of patients with TP (92%) had an unfavorable pattern of rADC change following therapy, as the rADC decreased between the two imaging time points. On the other hand, a majority of patients with PsP (86%) exhibited an increase in rADC change. Furthermore, in comparison to other imaging parameters, only rADC was reported to be significantly higher in the treatment response group compared to the non-responsive TP group.

**Figure 6:**
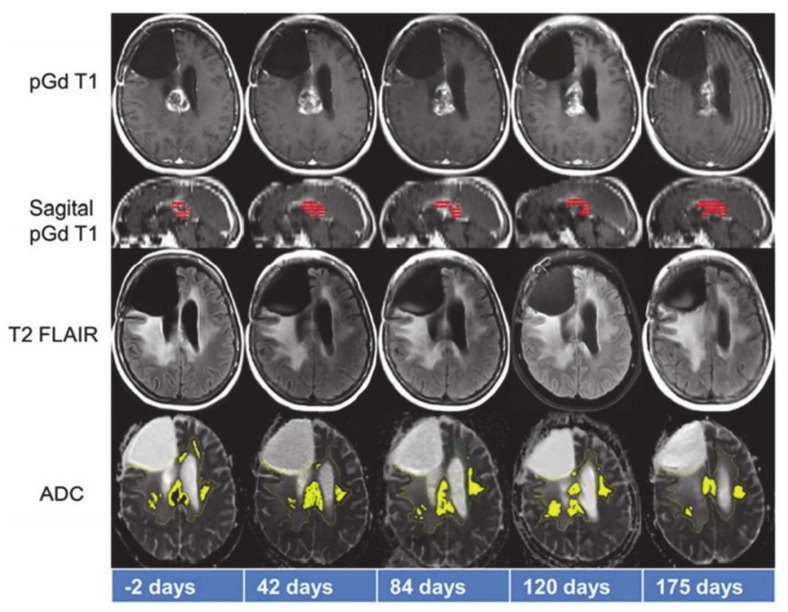
Representative anatomical images and apparent diffusion coefficient (ADC) maps from a patient who benefitted from treatment with anti-PD1 immunotherapy. While tumor volume on sagittal post-contrast T1 weighted images (red color) demonstrated initial declining trends from day 42–84, it increased from day 120–175. On the other hand, tumor volume as measured by considering that ADC values on the ADC maps (yellow color) decreased between day 120–175, indicating positive response. Reprinted with permission from ref. [95]. Copyright 2017 Springer-Verlag Berlin Heidelberg.

In another study, serial MR imaging was performed on recurrent GBM patients treated with anti-PD1 therapy with or without anti-CTLA-4 therapy to evaluate the potential of quantitative imaging in differentiating patients who derived therapeutic benefit from those who did not (Figure 6) [95]. Subsequent to subtraction of precontrast T1 weighted images from postcontrast T1 weighted images, the volume of interests (VOIs) representing the amount of abnormal tumor, based on the degree of contrast enhancement, were computed. Similarly, VOIs were drawn on hyperintense tumor abnormality on T2-FLAIR images to determine the FLAIR VOI. Subsequently, the FLAIR VOI was transposed onto the corresponding co-registered ADC maps. The volume of tissue within the FLAIR VOI having an intermediate ADC in the range of 0.7–1.1 × 10^−3^ mm^2^/s (IADC VOI) represented solid tumor tissue. The investigators of this study observed that IADC VOI decreased for all patients in the therapeutic benefit group despite demonstrating initial trends of IADC VOI increase. On the other hand, all patients in the non-benefit group demonstrated progressive increases in IADC VOI. The fact that therapeutic outcome was better correlated with ADC volume than with FLAIR abnormality or contrast enhancement volumes supports the notion that physiological imaging parameters may be more useful in evaluating treatment response to immunotherapy than conventional neuroimaging parameters. The investigators of this study also reported that patients deriving therapeutic benefit from immunotherapy were identified by ADC at an earlier stage (a median time of 93 days after treatment) than conventional MR imaging (a median time of 121 days after treatment).

Taken together, the results of these two studies signify ADC as a potential imaging to differentiate TP from immunotherapy-induced inflammatory response. In general, highly malignant regions of GBMs harboring closely packed tumor cells and reduced extracellular volume demonstrate low ADC values. It is hypothesized that successful immunotherapy would result in the accumulation of vasogenic edema and would reduce tumor cellularity, leading to increased ADC values. On the other hand, diffusivity of water molecules decreases in TP with high cellularity and reduced extracellular space. Anti-PD1 therapy is known to activate effector and cytotoxic T cells, leading to secretion of cytokines, which in turn causes tumor regression and also decreases the proliferation and infiltration of regulatory T cells within the tumor [46,98]. It is hypothesized that an increase in ADC values in GBMs following anti-PD1 immunotherapy is due to decreased tumor cellularity. Additionally, studies from a variety of tumor types [99,100] have revealed that increases in ADC reflecting therapy-induced apoptosis or necrosis of tumor cells precede gross morphological changes such as reduction in tumor size, suggesting ADC as a valuable parameter to evaluate early response to immunotherapy. 

While the diagnostic potential of FA in evaluating treatment response to ICIs has not been examined, a study found low FA values from peritumor regions of brain metastases where CD3^+^ T cells count was high (indicating low tumor cell density) [101]. Moreover, increased peritumoral CD3^+^ T cell density was significantly associated with favorable survival outcomes. Considering these observations, it would be interesting to see if future studies involving the use of FA along with other diffusion MR imaging-derived parameters can further assist in assessing treatment response to immunotherapies. 

Several studies have reported the utility of perfusion-weighted DSC-PWI and DCE-MRI-derived parameters in distinguishing TP from PsP in GBM patients treated with CCRT, indicating that these parameters may also be useful in evaluating treatment response to immunotherapies. A DCE-MRI study [102] from a GBM model of rats treated with natural killer cells reported decreased v_e_ from progressive tumors and increased v_e_ from tumors with reduced proliferation. However, contrary to the working hypothesis, Song et al. [96] did not observe any significant differences in absolute values or even in interval changes for perfusion parameters (rCBV, K^trans^, v_p_, and v_e_) between TP and PsP patients treated with anti-PD1 therapy. While cytotoxic effects of CCRT are known to cause fibrinoid necrosis, endothelial injury and occlusion of blood vessels [103], results of the study conducted by Song et al. [96] suggest that immune-related response induced by anti-PD1 therapy does not impact tumor hemodynamics in a similar fashion or to the degree that can be estimated by perfusion MR imaging. Another possible reason for the absence of significant differences might be related to confounding effects of anti-vascular bevacizumab, which was administered to some patients before the commencement of anti-PD1 therapy.

**Figure 7:**
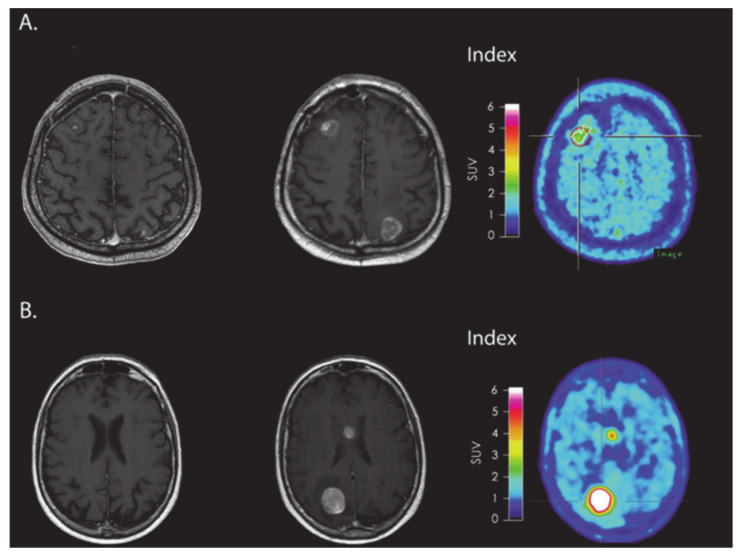
(**A**) MRI and FET-PET images of a patient with melanoma brain metastasis, diagnosed with PsP using immune-related response criteria (irRC) after receiving immune checkpoint inhibitor immunotherapy. The index MRI shows >25% increase in contrast-enhancing lesions located in frontal and occipital regions. Low metabolic tumor activity was observed on FET-PET images. (**B**) MRI and FET-PET images of a patient with melanoma brain metastasis, who was diagnosed with TP using irRC. The index MRI shows >25% increase in contrast-enhancing lesions located in the body of corpus callosum and occipital regions. A very high metabolic tumor activity was observed on FET-PET images. Reprinted with permission from ref. [109]. Copyright 2016 Oxford University Press.

Recently, some investigators have also explored other imaging modalities in evaluating treatment response in brain tumor patients. In a prospective study using FLT-PET imaging, Brahm et al. [104] did not observe any differences between TP from PsP in GBM patients who were treated with CCRT, mainly because of the fact that FLT uptake in GBMs reflects not only trapping of FLT in proliferating neoplastic cells but also disruption of BBB integrity [105]. The limited diagnostic utility of FLT–PET in distinguishing TP from PsP might be attributed to the fact that BBB leakage is known to occur in both TP and PsP. On the other hand, amino acid-based PET imaging tracers can cross the intact BBB, which allows the depiction of tumor regions beyond the contrast enhancement that is seen on MR imaging [106]. Using [^18^F]-FET as a PET imaging tracer, Kebir S et al. [26] were successful in distinguishing TP from PsP in GBM patients treated with CCRT. The plausible explanation might be that active tumor cells express higher concentrations of mobile protein and peptide components providing a higher contrast in TP than in PsP [107]. The other potential significance of using [^18^F]-FET-PET has been that FET tracer exhibits high uptake by neoplastic cells and less uptake by inflammatory cells [108]. Expanding on their previous work, Kebir S et al. [109] sought to assess the ability of FET-PET in evaluating treatment response to immunotherapy. In this small study, which included five patients with melanoma brain metastases who underwent ICI treatment (ipilimumab or nivolumab) at the time of the initial increase of brain tumor burden, the investigators found considerably higher metabolic activity in TP compared to PsP patients as measured by maximum tumor-to-brain ratio of FET-PET signal (Figure 7). These preliminary findings suggest that FET-PET PET provides potentially valuable information about tumor biology and presents an alternative diagnostic tool to assess treatment response to ICIs in brain tumor patients. However, these results should be interpreted with caution, and future studies with larger patient cohorts are required to confirm these initial findings.

### 4.2. Active Immunotherapy

**Figure 8:**
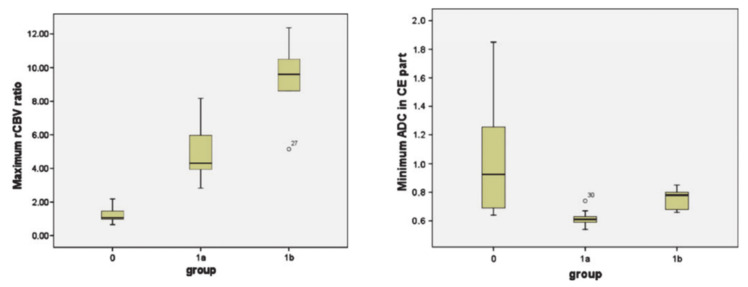
Box-and-whisker plots demonstrating the distributions of maximum relative cerebral blood volume (rCBV) ratios (left panel) and minimum ADC (right panel) from three groups (group 0: patients who remained stable during the follow-up period; group 1a: patients who were suspected but not confirmed with TP; group 1b: patients who were definitive TP). The bottom and top edges of boxes represent the 25th percentile and the 75th percentile values. The bands within the boxes represent 50th percentile (median) values. Whiskers display the range of data distribution. Outliers are marked with open circles. Reprinted with permission from ref. [110]. Copyright 2010 Springer-Verlag.

While evaluating treatment response to DC immunotherapy in a case series of eight recurrent GBM patients, Vrabec et al. [110] divided 32 follow-up MR imaging examinations into three groups (group I: patients who remained stable during the follow-up period; group II: patients who were suspected but not confirmed with TP; and group III: patients who were definitive TP). The investigators of this study found similar imaging patterns on post-contrast T1 weighted images and T2-FLAIR images. However, physiological MR imaging parameters demonstrated significantly higher minimum (ADC_min_) values in group I than in group II patients. Additionally, significantly higher maximum relative CBV (rCBV_max_) values were observed in tumors from group III compared to those from patients in group II as well as from patients who remained stable during the course of treatment (group I) (Figure 8). Notably, these results are different from those reported by Song et al. [96], where no significant differences in rCBV between TP and PsP patients treated with anti-PD1 therapy were observed. The difference in imaging findings between the two studies might be due to individual or combined effects of different types of immunotherapies, patient populations, or data acquisition and analytical approaches used. Despite this, results from Vrabec’s study indicate that rCBV_max_ and ADC_min_ parameters may be useful in the follow-up evaluation of GBM patients treated with DC immunotherapy.

An earlier study [111] also reported the benefits of using DSC-PWI in evaluating treatment response to tumor cell vaccine in a cohort of GBM patients who were previously treated with surgery and radiotherapy. After vaccine therapy, low rCBV areas corresponded to increasingly contrast-enhancing regions (mismatch areas) in three of six patients. Overall, patients treated with vaccines had longer survival than control patients; however, rCBV values did not correlate with treatment or with survival outcome measures. It is speculated that the mismatch regions in these three patients might have been due to infiltration by inflammatory cells. This hypothesis is supported by histopathological findings in immune-treated brain metastases showing reactive astrocytosis and scattered inflammatory and microglial cells surrounding isolated clusters of tumor cells [112].

In another study, Ceschin et al. [113] used DWI-derived parametric response maps (PRM) to distinguish TP from PsP in pediatric patients with diffuse intrinsic pontine glioma. All patients were treated with peptide-based vaccine therapy. The investigators observed a significantly higher fractional increase in ADC in PsP compared to TP patients. Additionally, the PRM, ratio defined as fractional increase in ADC /fractional decrease in ADC, was also higher in PsP compared to TP patients. However, there were no significant differences in fractional decrease in ADC, mean ADC or tumor volume between TP and PsP patients. Moreover, PsP patients (19.1 months) had longer median survival from the time of diagnosis than TP patients (12.5 months).

**Figure 9:**
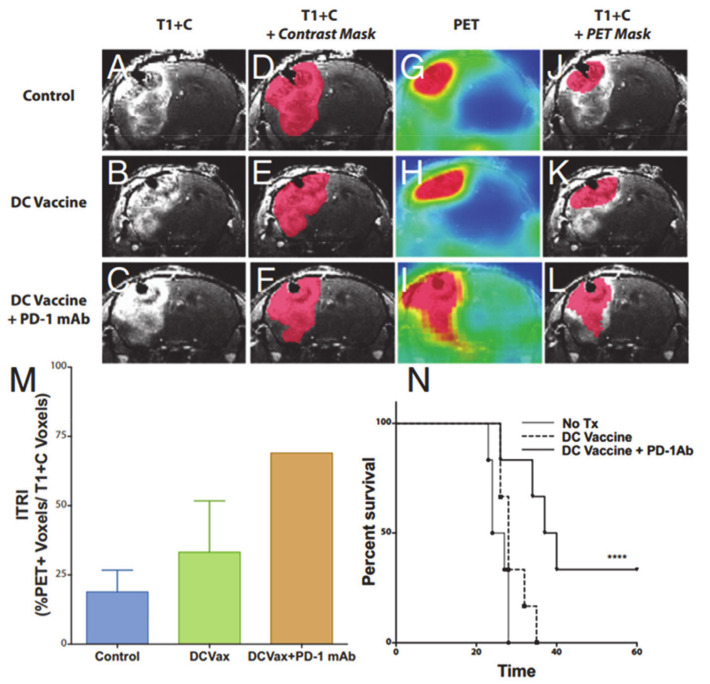
Representative coronal post-contrast T1 weighted images of untreated control, dendritic cell vaccination (DCVax; Northwest Biotherapeutics)-treated, and DCVax + PD-1 mAb-treated GBM bearing mice (**A**–**C**). Representative contrast subtraction maps (red; contrast mask) overlaid onto post-contrast T1 weighted images (**D**–**F**). Representative coronal [^18^F]-FAC PET images of untreated control and DCVax- and DCVax + PD1 mAb-treated mice (**G**–**I**). Representative threshold PET subtraction maps (red; PET mask) overlaid onto post-contrast T1 weighted images (**J**–**L**). The immunotherapeutic response index (ITRI, a ratio of the PET voxels divided by the T1+C subtraction voxel data) calculated for each treatment group is shown (**M**). Increased ITRI values were observed in mice treated with DCVax and/or PD-1 mAb compared with untreated mice. Survival plots of intracranial GBM-bearing untreated control (no Tx), DCVax-treated, and DCVax + PD-1 mAb-treated mice (**N**). Reprinted with permission from ref. [114].

**Figure 10:**
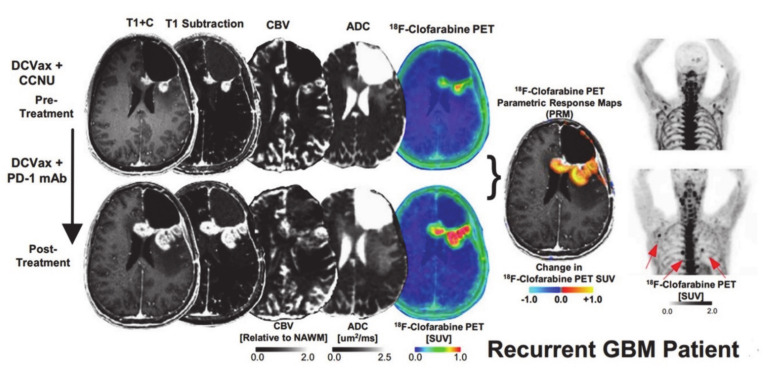
Post-contrast T1 weighted, T1-subtraction, rCBV, ADC, [^18^F]-FAC PET + MRI fusion and whole-body maximum intensity projection images of [^18^F]-CFA from a patient with recurrent GBM before (top) and after (bottom) immunotherapy are shown. Reprinted with permission from ref. [114].

In a study by Antonios et al. [114], PET probes for deoxycytidine kinase (dCK) were evaluated in both mice and patients with GBM undergoing treatment with immunotherapy (Figure 9 and Figure 10). In an orthotopic malignant glioma model, the animals were treated with DC vaccination and/or anti-PD-1 therapy. Mice then underwent [^18^F]-2-fluoro-d-(arabinofuranosyl) cytosine ([^18^F]-FAC) PET imaging and post-contrast MR imaging. [^18^F]-FAC is a deoxycytidine analog that is a specific substrate for dCK and is specially taken up in activated cytotoxic T cells indicating regions of immune cell activity. The authors reported significantly increased [^18^F]-FAC uptake in DC vaccinated + anti-PD1-treated mice compared to DC-vaccinated or untreated control mice. The imaging finding was in agreement with immunohistochemical analysis showing the maximum population of infiltrating T-lymphocytes in the DC-vaccinated + anti-PD-1 therapy treated mice followed by DC vaccinated than control animals. Not surprisingly, post-contrast MR images demonstrated comparable tumor growths in glioma-bearing animals, suggesting that standard MR imaging is not effective in distinguishing tumor growth from antitumor response induced by immunotherapies. When the preclinical work was extended to evaluate treatment response in three GBM patients receiving tumor lysate-pulsed DC vaccine with or without pembrolizumab, post-treatment PET images demonstrated enhanced tumor uptake of [^18^F]-CFA. However, tumor volumes remained constant before and after treatment. Due to the preliminary nature of these findings, additional studies are required to understand the significance of [^18^F]-CFA-PET findings in GBM patients receiving immunotherapeutic treatments.

### 4.3. Adoptive Immunotherapy

**Figure 11:**
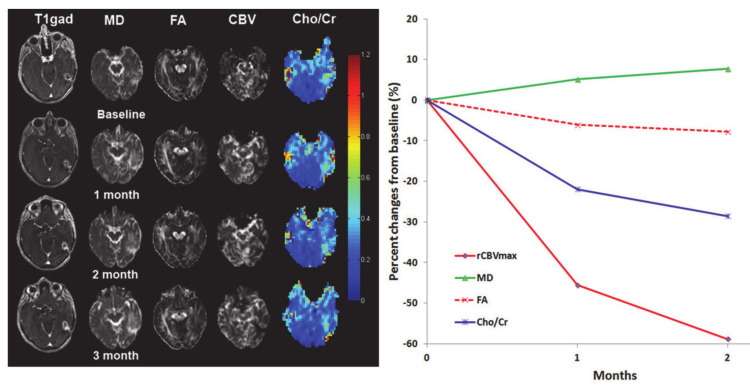
Representative baseline and follow-up anatomical images and parametric maps at baseline and follow-up periods from a patient treated with epidermal growth factor receptor deletion mutation (anti-EGFRvIII) chimeric antigen receptor T cell therapy. Percentage changes in parameters from baseline to 1-, and 2-month follow-up periods from this patient are shown. Reprinted with permission from ref. [115]. Copyright 2019 Springer Nature.

Treatment response to CAR-T cell therapy from 10 recurrent GBM patients was evaluated at 1, 2 and 3 month follow-up periods using MR methods (Figure 11) [115]. In this study, all tumors exhibited increased volume relative to baseline on post-contrast T1-weighted images secondary to the presence of pronounced inflammatory response, making it difficult to assess therapeutic response, based on conventional imaging. However, when percentage changes in DTI and DSC-PWI-based parameters were assessed, no definite trends in imaging parameters were observed in most of the patients, indicating that if used in isolation, these physiologic parameters may have a limited role in assessing treatment response to EGFRvIII CAR-T cell therapy.

In another study from this group [31], a multiparametric MRI approach demonstrated a more accurate assessment of treatment response. A classification model was developed in this study to predict the progression probability (PP) by using different imaging parameters to evaluate treatment response in GBM patients who had received standard treatment (surgery and CCRT). A combination of FA, CL and rCBV_max_ from the contrast-enhancing regions were able to differentiate TP from PsP with an accuracy of 90%. While evaluating treatment response to EGFRvIII CAR-T cell therapy in GBM patients in another study [115], the PP values derived from the predictive model were shown to objectively and correctly characterize each lesion as either TP or PsP at each individual time point. These promising findings suggest that combined use of DTI and DSC-PWI may provide more accurate assessment of treatment response in GBM patients treated with EGFRvIII CAR-T cell therapy than an individual parameter or technique. Since adoptive immunotherapy is an emerging modality to treat recurrent GBMs, clinical trials with larger patient populations and multiple clinical end points are required to establish the usefulness of PP values in evaluating treatment response to EGFRvIII CAR-T cell therapy.

Several studies have reported the clinical potential of proton MR spectroscopy (^1^H MRS) for studying brain tumor metabolism [116,117] and evaluating treatment response [17,118,119,120,121] in brain tumor patients treated with CCRT and anti-angiogenic agents. Collectively, these studies have demonstrated significantly increased choline/N-acetylaspartate (NAA) and/or Cho/creatine (Cr) ratios from solid/contrast-enhancing regions of tumors in patients with recurrent tumors and TP than those with radiation necrosis and PsP. However, these studies used single-voxel or single-slice multivoxel ^1^H MRS methods that are usually constrained by limited spatial coverage of the tumor. In contrast, three dimensional (3D)-echo planar spectroscopic imaging (EPSI) sequence provides volumetric metabolite maps with high spatial resolution [122]. Using 3D-EPSI, a recent study [123] reported significantly higher Cho/Cr from contrast-enhancing regions and Cho/NAA from contrast-enhancing as well as from peritumor regions of tumors in TP than those with PsP. These findings suggest that mapping of metabolite ratios from peritumoral regions should also be considered when evaluating treatment response, which is only possible when a 3D-^1^HMRS sequence is used. Despite promising findings, ^1^H MRS has not been widely used in the assessment of treatment response to immunotherapy in GBMs. In a solitary study, Wang et al. [115] analyzed 3D-EPSI data from contrast-enhancing regions of tumors in patients with recurrent GBM treated with EGFRvIII CAR-T cell therapy (Figure 11). The investigators reported decreasing trends in some patients as well as increasing and variable trends in other patients in Cho/Cr ratio at follow-up periods relative to baseline, implying that ^1^H MRS can be a useful tool for evaluating treatment response to immunotherapies in GBMs.

To optimize the therapeutic potential of CAR T cell immunotherapy, it is also important to monitor the trafficking, biodistribution and viability of immune cells within the tumor site. In this effort, reporter gene-based PET imaging is being explored in monitoring trafficking, targeting and activation of therapeutic cells in tumors [124,125]. Keu et al. [126] used PET imaging with 9-[4-[^18^F] fluoro-3-(hydroxymethyl) butyl] guanine ([^18^F]-FHBG) to track IL13 Rα2 CAR T cells expressing a wild-type HSV1-tk reporter gene in a population of seven GBM patients. Each patient underwent [^18^F] FHBG PET imaging before and after infusion of CAR T cells. Moreover, this method demonstrated the presence of a variable degree of infiltrated cytotoxic T-lymphocytes expressing CARs targeting IL13Rα2 antigens in the tumor regions. Further optimization of this imaging approach for monitoring in vivo cell trafficking may greatly benefit cell-based immunotherapies for GBMs.

### 4.4. Oncolytic Viral Therapy

To date, no study has been conducted to evaluate treatment response to oncolytic viral therapies using physiologic MR imaging techniques in GBM patients. However, molecular imaging using PET and single photon emission computed tomography (SPECT) probes have been used to monitor tumors infused with oncolytic viruses and viral-mediated gene therapy through HSV type-1-tk gene (HSV-1-tk) reporter gene. In a PET study with ^124^I-labelled 2′-fluoro-2′-deoxy-1-D-arabino-furanosyl-5-iodo-uracil, ([^124^I]-FIAU) was used as a specific marker for gene expression of HSV-1-tk to identify the location, magnitude and extent of vector-mediated HSV-1-tk gene expression in a phase I/II clinical trial of gene therapy in recurrent GBM patients [127]. The investigators of this study found that transduction of HSV-1-tk gene with subsequent prodrug activation by ganciclovir was safe with no or minimal occurrences of adverse events, but the clinical response of the therapy was poor. Similarly, a study [128] using the SPECT tracer ^124^I-FIAU demonstrated the feasibility in a clinical setting but could not provide any evidence of accumulation of the reporter probe in tumors treated with an oncolytic HSV. Collectively, these preliminary studies indicate that molecular imaging techniques may be able to monitor viral replication in GBM. Despite these promising studies, it is not yet clear whether these techniques can be used to evaluate response to oncolytic viral immunotherapy in GBM patients.

## 5. Concluding Remarks and Future Perspectives

Despite technical challenges, physiological MR imaging techniques provide quantifiable, unbiased and physiologically relevant information in the post-therapeutic characterization of GBMs treated with immunotherapy. Given that diffusion and perfusion MR imaging-derived parameters reflect inherently different physiological processes, they provide complementary information about the tumor microenvironment. As such, combined analysis of diffusion and perfusion MR imaging parameters may provide greater accuracy in evaluating treatment response to immunotherapies in GBM patients.

Diffusion and perfusion MR imaging techniques have already been incorporated in routine clinical imaging protocols in a large number of clinical academic centers worldwide. However, the acquisition protocol is highly variable among imaging centers, and variability in imaging protocols as well as analytical methods can impact the generalizability of imaging techniques across different sites. Fortunately, standardized protocols have been proposed specifying the acquisition parameters for diffusion [129] as well as perfusion MR imaging [130] sequences. Wider adoptions of these standardized protocols by the neuro-oncology community will aid in validating and establishing the potential of these physiologic imaging-derived parameters in the evaluation of treatment response to immunotherapy in GBM patients.

PET imaging also provides important functional information about the tumor microenvironment and has been useful in evaluating treatment response to immunotherapies in GBMs. An important component of immunotherapies is the presence of profound inflammation at the tumor bed that presents a significant challenge in the accurate assessment of treatment response. PET imaging probes that selectively accumulate in immune cells rather than in neoplastic cells may be valuable in the therapeutic assessment. In this direction, potential utility of PET imaging probes such as translocator protein (TSPO) [131] and (S)-2-amino-3-[3-(2-^18^F-fluoroethoxy)-4-iodophenyl]-2-methylpropanoic acid (^18^F-FIMP) [132] targeting neuroinflammatory cells (lymphocytes and macrophages) is actively being investigated. Other potential approach to address the problem of neuroinflammation is to monitor trafficking and activity of effector immune cells within the tumor beds using imaging techniques. Towards that end, PET imaging agents based on antibodies or antibody derivatives that target effector immune cells are currently being evaluated in clinical trials. In such a study [133], patients with metastatic solid tumors undergoing checkpoint inhibitor therapy underwent PET imaging with anti-CD8 radiolabeled minibody (Mb) ^89^Zr-IAB22M2C. Preliminary data indicated that CD8 T cells in tumors were detected in tumors by 24 hours post-infusion, suggesting the usefulness of this new PET tracer in tracking immune cells in tumors.

With the advent of integrated PET-MR imaging scanners, we believe that simultaneous acquisition and analysis of co-registered PET and MR imaging data providing complementary physiologic information will allow more accurate assessment of treatment response to immunotherapies in GBMs than a single imaging modality alone. Besides treatment evaluation, there is an urgent need for the development of objective, reliable and quantitative prognostic imaging biomarkers to facilitate treatment stratification and selection of GBM patients for enrollment in various immunotherapeutic trials. In this direction, a newer generation of molecular PET imaging tracer (zirconium-89 labeled atezolizumab) is currently under investigation to predict response to anti-PD-LI immunotherapy and upfront patient selection in extracranial cancers [134]. We believe that similar PET tracers will be developed to predict response to various immunotherapeutic regimens in GBMs.
